# Cannabis Use in Autism: Reasons for Concern about Risk for Psychosis

**DOI:** 10.3390/healthcare10081553

**Published:** 2022-08-16

**Authors:** Riccardo Bortoletto, Marco Colizzi

**Affiliations:** 1 Child and Adolescent Neuropsychiatry Unit, Maternal-Child Integrated Care Department, Integrated University Hospital of Verona, 37126 Verona, Italy; 2 Department of Psychosis Studies, Institute of Psychiatry, Psychology and Neuroscience, King’s College London, London SE5 8AF, UK; 3 Unit of Psychiatry, Department of Medicine (DAME), University of Udine, 33100 Udine, Italy

**Keywords:** neurodevelopment, schizophrenia, prevention, mental health services, cannabinoids

## Abstract

Being particularly vulnerable to the pro-psychotic effects of cannabinoid exposure, autism spectrum individuals present with an increased risk of psychosis, which may be passed on to their own children. More specifically, cannabis exposure among autism spectrum individuals seems to exert disruptive epigenetic effects that can be intergenerationally inherited in brain areas which play a critical role in schizophrenia pathophysiology. Additionally, because of such cannabinoid-induced epigenetic effects, autism candidate genes present with bivalent chromatin markings which make them more vulnerable to subsequent disruption, possibly leading to psychosis onset later in life. Thus, findings support a developmental trajectory between autism and psychosis, as per endocannabinoid system modulation. However, such evidence has not received the attention it deserves.

## 1. Setting the Scene

Prevention and early intervention may have a remarkable impact on youth mental health and quality of life. On one hand, acting on determinants of mental health in the whole community (e.g., nutrition and physical health, housing, family support, child maltreatment, stressful life events coping strategies, education and schooling, addictive substance misuse/dependence) may help promote psychological wellbeing. On the other hand, screening procedures at the universal, selective, and indicated level can help detect situations of clinical vulnerability prior to the onset of a frank psychiatric disorder, with the aim of improving their outcome by halting their progression or blunting the severity of the disease course. In fact, when the clinical picture deteriorates into a full-blown psychiatric disorder, there is still room for prevention, with a referral to multilevel early intervention services potentially softening factors that may lead to a worse prognosis [1].

## 2. A Clinically Relevant Nosographic Change

To this extent, the conceptual and clinical shift from “prodrome” to “at-risk mental state (ARMS)”, when referring to the phases preceding the development of schizophrenia spectrum disorders (and, broadly speaking, mental disorders), took the limelight towards the end of last century and is a highly debated topic to this day. Indeed, the former concept refers to attenuated symptoms underlying an already inevitable progress to an overt disease; instead, the “ARMS” concept provides a less deterministic key to understanding schizophrenia, in favor of a psychosis-spectrum view in which milder manifestations can be found and where early intervention is still possible [1].

Clinical high-risk for psychosis (CHR-P) individuals present with low-threshold psychotic symptoms, impairments encompassing cognition, global functioning, and social–emotional intelligence, along with frequently associated comorbidities. Recent meta-analytic evidence concerning adolescents and youth in the general population, as well as from clinical populations, shows a CHR-P prevalence more than tenfold higher in the clinical groups (1.7% vs. 19.2%) [2]. These findings are coherent with the evidence that the risk enrichment, and thus the transition risk for psychosis eventually observed, is low in the community, but higher in secondary mental health services. With one in five young people screened in clinical contexts meeting the CHR-P criteria, the importance of watchful detection and management tools is very clear.

## 3. Cannabinoids in the Neurodevelopmental Continuum

Among the risk factors precipitating the onset of psychosis and worsening its outcome in adolescence and youth, substance use certainly plays a major role, and is observed to be highly prevalent both in young people with first episode of psychosis (FEP) and in subjects to be considered at clinical high risk. Cannabis is the most used recreational substance worldwide among adolescents and can produce detrimental effects on brain maturation in the pubertal period, a crucial phase for sensitization of the dopaminergic system [3]. As such, high levels of Δ-9-tetrahydrocannabinol (THC) through cannabis use increase susceptibility to impaired learning and memory recall [4], negative and positive psychotic symptoms [5], as well as the risk of developing full-blown psychosis [6]. Early, more frequent, and/or continuous high-potency cannabis exposures are associated with an augmented likelihood of developing subtle psychotic symptoms or FEP, with lifetime cannabis use being associated with poorer outcomes [7]. Additionally, smoking mixtures containing synthetic cannabinoids (SCs) have flooded the market for the last 15 years, becoming increasingly popular among people willing to experience a cannabis-like high (e.g., Spice, K2, Yucatan Fire). It soon became clear that even moderate doses of SCs may produce significant severe psychotomimetic adverse events [8]. Possible explanations for the relationship between cannabis use and psychosis have been debated for a long time, with more solid evidence of a direct effect of cannabis use in leading to the onset of psychosis and impacting on its progression, and more nuanced evidence of an inverse effect where psychosis would make the person more prone to use cannabis in an attempt to self-medicate [9].

Intriguingly, a unifying hypothesis suggests that cannabis use and psychiatric disorders, especially psychosis, notably share common neurobiological underpinnings [10]. Interestingly, schizophrenia may be preceded by neurocognitive and developmental difficulties in early childhood, and common psychotic symptoms may be phenotypically similar to manifestations occurring in autism spectrum disorders (ASDs), such as impaired social–communicative skills, repetitive stereotyped behaviors, and perceptual abnormalities [11]. In turn, autism has been widely described as a lifelong developmental disorder, whose many fundamental deficits exhibited during toddlerhood tend to improve over time, especially in less verbally compromised individuals; however, this does not indicate an evolution towards milder forms of autism, but rather a qualitative shift in the pattern of traits displayed [12]. To this end, neurodevelopmental disorders (NDDs) might be considered as part of an evolutionary trajectory that embraces, at its extreme, mental health disorders arising in late adolescence and young adulthood, such as affective and non-affective psychoses [13].

To further explore the above-mentioned neurodevelopmental continuum paradigm, we recently conducted a systematic review aimed at investigating the puzzling connection between autism and psychosis through the contribution of endocannabinoid (eCB) system modulation [14]. Overall, evidence from preclinical and clinical studies included in the systematic analysis converges on an overlapping relationship between autism and psychosis [15,16], with a potential developmental trajectory from autism to psychosis as a function of the eCB system disruption. Little evidence emerged of a diametral association between autism and psychosis, as originally stated in the 1980s [17,18]. Instead, correction of eCB system alterations was demonstrated to be crucial to mitigate several autism and schizophrenia features, thus suggesting that its modulation may provide interesting opportunities for novel treatment strategies and corroborate the possibility of a dimensional therapeutic approach for both conditions [18]. Additionally, the effects of endocannabinoid-based treatments appeared to rely on epigenetic changes across genes known to be involved in key mechanisms underlying both autism and schizophrenia [19], as well as to the shared genetic vulnerability between autism, schizophrenia, and cannabis use [20,21]. Regarding the latter, autism-related genes have been suggested to increase the liability for lifetime cannabis use [20], which in turns fuels the developmental cascade to psychosis [9]. Additionally, psychotic experiences among the general population have been reported to be sustained by genetic vulnerability for ASD, and are also more distressing in the context of a genetically perturbed eCB system [22]. Most importantly, despite some inconsistent animal and human evidence that higher-dose THC exposure may be ameliorative for behavioral disturbances in autism, the overwhelming majority of data highlight how THC/cannabis use deeply affects brain functioning and alters autism- and schizophrenia-related pathways genes [23], leading to schizophrenia-like phenotypes [18,24] and related biological alterations [25,26] in ASD individuals, possibly transitioning through an ARMS [24]. Specifically, THC exposure produces disruptive methylation effects involving several neurodevelopment- and autism-related genes [25,26], with two main implications. Firstly, the altered methylation pattern is shown to be intergenerationally inherited in brain areas, such as the nucleus accumbens, which plays a critical role in schizophrenia pathophysiology [25]. Secondly, autism candidate genes present with bivalent chromatin markings because of THC-induced methylation effects, and are more prone to subsequent disruption, possibly leading to psychosis onset later in life [26]. In other words, autistic individuals seem to show remarkable vulnerability to the pro-psychotic effects of cannabinoid exposure, which may, in addition, be transmitted to their progeny, further supporting the validity of overlapping and developmental trajectory models between autism and psychosis, as per the eCB system modulation.

## 4. Time to Broaden the “ARMS” Group?

Two considerations follow from the evidence summarized so far. First, when we speak of ARMS, a narrowed approach to the notions of psychosis “risk” and “transition”, based on positive psychotic symptom manifestation alone, has failed to guarantee a satisfactory detection rate, currently estimated at approximately 5% [27]. Second, for years we have searched through presumably highly specific bio-behavioral risk factors for schizophrenia (e.g., dopamine system dysregulation [28], schizotypal personality [6]) for the reasons why some individuals are intrinsically more vulnerable to cannabis-induced psychosis, with findings that are not always unequivocal. In this regard, the risk of psychosis among autism individuals and its cannabis-induced amplification effect have been dramatically overlooked. Indeed, while the risk of cannabis-induced psychotic experiences, FEP, and transition to psychosis have been widely explored in CHR-P cohorts [29] and otherwise healthy adolescents and youth [30,31], to the best of our knowledge, longitudinal studies designed to assess the precipitating effects of earlier, heavier, and/or longer-term cannabis misuse over ASD individuals are totally lacking. Should psychiatrists extend the “at-risk mental state” for psychosis paradigm to ASD?

## 5. Raising Concern about Cannabis Exposure in Autism Spectrum Disorder

Hand in hand, cannabinoid-based medications have become increasingly popular in modern medicine, showing promising results in terms of efficacy and tolerability, sometimes comparable to conventional pharmacotherapy. To date, the FDA has approved purified cannabidiol (CBD), nabiximols, dronabinol, and nabilone with indications for treatment in very specific neuropsychiatric conditions [32]. In parallel, great interest has been aroused regarding the therapeutic potential of terpenoids and endocannabinoid-like compounds [33,34].

Medical cannabis is usually warmly welcomed (and demanded) by the families of young treatment-resistant ASD patients, often driven by evidence of CBD as a successful treatment for ASD-related symptoms and comorbidities (e.g., Dravet syndrome, Rett syndrome, Lennox–Gastaut syndrome), and as a somewhat natural product, devoid of any adverse effects. However, the occurrence of adverse outcomes is unclear due to the wide range of cannabis-based medications’ compositions and dosages within the studies [18,35,36]. Currently, there is a paucity of preliminary mixed findings examining their safety and efficacy as a treatment for ASD, warranting the need for larger-scale controlled studies to be performed. As a consequence, such prescriptions call for caution, and mental healthcare professionals should be concerned about the possibility of progression between ASD and schizophrenia precipitated by excessively high THC concentrations exposure. More importantly, public education is needed to warn against recreational cannabis use in ASD adolescents and youth, providing them with suitable information about their vulnerability of progression to a major psychiatric disorder. Early intervention clinics for people at risk of psychosis could benefit from the collaboration of professionals with different expertise, in order to intercept ASD subjects at an early stage of psychosis and offer preventive measures to protect them from environmental factors, such as cannabis use, that can deteriorate their mental health status.

## Data Availability

Not applicable.
